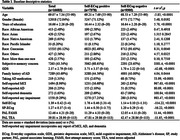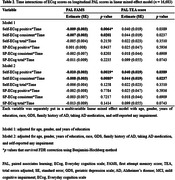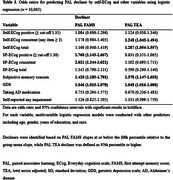# Everyday Cognition Scale Predicts Longitudinal Progression in Paired Associated Learning: Unsupervised Online Setting from the Brain Health Registry

**DOI:** 10.1002/alz.094622

**Published:** 2025-01-09

**Authors:** Jaemyeong Kang, Manchumad Manjavong, Chengshi Jin, Adam Diaz, Miriam T. Ashford, Joseph Eichenbaum, Emily Thorp, Elizabeth Wragg, Kenton Zavitz, Francesca K Cormack, Anna Aaronson, Melanie J. Miller, Scott R Mackin, Rachana Tank, Bernard Landavazo, Diana Truran‐Sacrey, Sarah Tomaszewski Farias, Michael W. Weiner, Rachel L. Nosheny

**Affiliations:** ^1^ UCSF, San Francisco, CA USA; ^2^ Gil Medical Center, Gachon University College of Medicine, Incheon Korea, Republic of (South); ^3^ San Francisco Veterans Affairs Medical Center, San Francisco, CA USA; ^4^ University of California, San Francisco, San Francisco, CA USA; ^5^ Khon Kaen University, Khon Kaen Thailand; ^6^ Veterans Affairs Medical Center, San Francisco, CA USA; ^7^ Northern California Institute for Research and Education (NCIRE), San Francisco, CA USA; ^8^ Cambridge Cognition, Cambridge United Kingdom; ^9^ Cambridge Cognition, Cambrdige, MA USA; ^10^ University of Cambridge, Cambridge United Kingdom; ^11^ NCIRE Northern California Institute for Research education, San Francisco, CA USA; ^12^ Center for Imaging of Neurodegenerative Diseases, San Francisco Veterans Administration Medical Center, San Francisco, CA USA; ^13^ Mental Health Service, Department of Veterans Affairs Medical Center, San Francisco, CA USA; ^14^ University of California, San Francisco Department of Psychiatry, San Francisco, CA USA; ^15^ Dementia Research Center UCL Institute of Neurology University College London, London United Kingdom; ^16^ University of California, Davis School of Medicine, Sacramento, CA USA; ^17^ VA Advanced Imaging Research Center, San Francisco Veterans Affairs Medical Center, San Francisco, CA USA

## Abstract

**Background:**

Online platforms are an efficient means to detect early cognitive decline, but few studies have investigated the relationship between remotely collected subjective cognitive change and cognitive decline. We hypothesized that the Everyday Cognition Scale (ECog), a subjective change measure, predicts longitudinal change in cognition in Brain Health Registry (BHR), an online registry for neuroscience research.

**Method:**

From the BHR database, we included participants aged 55+ who completed both the baseline ECog and repeated administrations of the CANTAB® Paired Associates Learning (PAL) test. Both self‐reported ECog (Self‐ECog) and study partner ECog (SP‐ECog), and two PAL scores (first attempt memory score [FAMS] and total errors adjusted [TEA]) were assessed. We used multiple ECog scoring outputs, based on previously established cut‐offs for likely impairment (Self‐ECog positive [total score ≥1.31], SP‐ECog positive [total score ≥1.36], and ECog consistent [any item≥3]). A linear mixed effects model was employed to assess the effect of baseline ECog on longitudinal change in PAL. Additionally, logistic regression models were used to assess the ability of ECog to identify ‘decliners’, who exhibited the worst PAL progression slopes corresponding to the fifth percentile and below.

**Result:**

The study included a total of 16,683 participants, who were followed for 11.49±11.53 months. Both Self‐ECog positive (estimate = ‐0.01, *p*<0.0019) and Self‐ECog consistent (estimate = ‐0.008, *p*<0.0085) were significant predictors for longitudinal change in PAL FAMS after adjusting for age, gender, education, race, depression, family history of Alzheimer’s disease (AD), taking AD medication, and self‐reported impairment. Those who were SP‐ECog positive (Odds ratio [95% confidence interval] = 1.760 [1.143–2.667]) and SP‐ECog consistent (2.021 [1.344–3.021]) had higher probability of being decliners based on PAL FAMS. Regarding the prediction of PAL TEA decliner, both Self‐ECog consistent (1.248 [1.043–1.494]) and Self‐ECog total (1.287 [1.054–1.557]) were associated with higher odds of being decliners.

**Conclusion:**

In the BHR’s unsupervised online setting, ECog demonstrated utility in predicting longitudinal progression in PAL scores, both in terms of continuous changes and when dichotomized as a decliner. Online, self‐administered measures of subjective cognitive change, together with objective neuropsychological test results have great potential to identify individuals with cognitive impairments.